# Supplementary material for: Cyclic Hypoxia Conditioning Alters the Content of Myoblast-Derived Extracellular Vesicles and Enhances Their Cell-Protective Functions
Source: Biomedicines. 2021 Sep 13;9(9):1211. doi: 10.3390/biomedicines9091211 (PMC8471008; doi:10.3390/biomedicines9091211)
Supplement: Supplementary file 1 [file biomedicines-09-01211-s001.zip › biomedicines-1348138-supplementary/supplementary material/supplementary methods tables figures.pdf]

Supplementary methods, tables and figures

Title: Cyclic Hypoxia Conditioning Alters the Content of Myoblast-Derived Extracellular Vesicles and Enhances Their Cell-Protective Functions

Authors: Yan Yan[1,2], Tingting Gu[3], Stine Duelund Kaas Christensen[4], Junyi Su[1], Thomas Ravn Lassen[5], Marie Vognstoft Hjortbak[5], IJu Lo[1], Susanne Trillingsgaard Venø[2], Andrea Erzsebet Tóth [6], Ping Song[1], Morten Schallburg Nielsen[6], Hans Erik Bøtker[5], Blagoy Blagoev[4], Kim Ryun Drasbek[3], Jørgen Kjems[1,7*]

1. Interdisciplinary Nanoscience Center, Aarhus University, Aarhus, Denmark. 2. Omiics ApS, Aarhus, Denmark. 3. Center of Functionally Integrative Neuroscience, Department of Clinical Medicine, Aarhus University, Aarhus, Denmark. 4. Department of Biochemistry and Molecular Biology, University of Southern Denmark, Odense, Denmark. 5. Department of Cardiology, Aarhus University Hospital, Skejby, Denmark. 6. Department of Biomedicine, Aarhus University, Aarhus, Denmark. 7. Department of Molecular Biology and Genetics, Aarhus University, Aarhus, Denmark.

*Corresponding author: Jørgen Kjems; Interdisciplinary Nanoscience Center, Aarhus University, Aarhus, Denmark; Department of Molecular Biology and Genetics, Aarhus University, Aarhus, Denmark. (jk@mbg.au.dk)

Supplementary methods

Mass spectrometry (MS) based proteomics of EVs

Protein sample MS preparation

The 10 tubes containing exosome pellets were thawed on ice and 8 M guanidine hydrochloride (dissolved in MilliQ water) was added to reach a final guanidine hydrochloride concentration of 6 M. The samples were kept at room temperature for the rest of the procedure. To reduce the proteins 2 mM DTT (dithiothreitol) was added and the samples were incubated for 30 mins. Afterwards, the proteins were alkylated using 11 mM CAA (chloroacetamide) and incubated in the dark for 30 min. The samples were diluted with 50 mM ABC (ammonium bicarbonate) to a final guanidine hydrochloride concentration of 2 M and a pH between 8 and 9. To digest the proteins, first LysC was added in a ratio of 1:100 (enzyme:protein) and incubated for 3h. The samples were then diluted with 50 mM ABC to a final guanidine hydrochloride concentration of 1 M, Trypsin was added at a ratio of 1.25:100 (enzyme:protein), and the samples were incubated over-night with gentle rotation.

High pH (HpH) reversed phase fractionation

To generate a spectral library for the DIA (data-independent acquisition) analysis, parts of the samples were fractionated using HpH reversed phase fractionation as previously described [1] with minor modifications. 45 % (around 22.5 μg) of sample from one N EV and one HR EV samples were mixed prior to HpH fractionation. Two C18 Empore disks (3M) were plugged in a p200 pipette tip and covered with approximately 1 cm of C18 beads (ReproSil-Pur, 1.9 μm, Dr. Maisch). This HpH Stagetip was activated, equilibrated and washed using 100 % methanol, HpH solvent B (50 % ACN (acetonitrile) adjusted to pH 10 with ammonium hydroxide) and two times with HpH solvent A (MilliQ water adjusted to pH 10 with ammonium hydroxide). The pH of the sample was adjusted to pH 10 using ammonium hydroxide as well, prior to loading on the Stagetip. The flow-through (FT) was collected. Using HpH solvent A the Stagetip was washed, and the wash FT was collected and combined with the previous FT. The peptides were eluted sequentially from the Stagetip column into 15 fractions using the following percentages of ACN: 1.75, 3.5, 5.25, 7, 8.75, 10.5, 14, 17.5, 21, 24.5, 28, 31.5, 35, 50 and 70%. Using a SpeedVac centrifuge the fractions

were evaporated leaving around 10-20 µL, and afterwards re-suspended in 100 µL Buffer A (0.6 % ACN + 0.1 % TFA (trifluoroacetic acid)).

For each fraction and the FT+wash, a STAGE-tip cleaning and concentration was made using a P200 pipette tip plugged with two C18 disks [1]. STAGE tips were activated, equilibrated and washed using 100 % methanol, Solvent B (50 % ACN/0.5 % Acetic Acid) and two times with Buffer A (0.6 % ACN/0.1 % TFA). The samples were loaded on the STAGE tips. The pH of the FT+Wash was adjusted to a pH below 3 using 10 % TFA prior to loading of the sample. STAGE tips were then washed using Buffer A and eluted with 2x25 µL Solvent B. The samples were evaporated in the SpeedVac centrifuge to a few µL and resuspended in Buffer A before being loaded on a 96 well plate.

The same STAGE tip purification protocol was used to clean 5% (around 2.5 µg) of each replicate of the two conditions prior to DIA analysis. In the wells with the samples 1:15 of iRT (Biognosys) retention time peptides was added.

LC-MS/MS analysis

High-accuracy mass spectrometric analysis were perform using an Easy-nLC 1000 system (Thermo Scientific) coupled to a Q Exactive HF-X mass spectrometer (Thermo Scientific) as described [2]. Separation of the samples by liquid chromatography (LC) was conducted using an 18 cm fused silica column (packed in-house) with 1.9 µm C18 beads (ReproSil-Pur, Dr. Maisch), heated to 50°C using a column heater.

For the HpH fractionated samples, a 120 minutes gradient was applied with an increasing concentration of Solvent B (80% acetonitrile, 0.5 % acetic acid) at a flowrate of 250 nL/min. The mass spectrometer was set to positive ion mode. The full MS scan resolution was 60000, had a scan range of 300-1700 m/z, an automatic gain control (AGC) target value of 3e6 and the maximum injection time (IT) was set to 15 ms. For MS/MS a data-dependent top-12 acquisition mode was used, with the resolution set to 60000, a maximum IT of 110 ms, and a scan range between 200 and 2000 m/z. The isolation window was set to 1.2 m/z, and the normalized collisional energy (NCE) was 28%. The dynamic exclusion was set to 45 seconds, and the charges 1, 8 and above were excluded from fragmentation.

For the DIA analysis of each replicate, a 120 minutes gradient with an increasing Solvent B concentration at 250 nL/min was applied as well. The mass spectrometer was in positive ion mode, full MS scan resolution was 60000, had a scan range of 350-1400 m/z, an AGC target value of 3e6 and a maximum IT of 45 ms. After each full scan 56 PRM spectra were acquired. These were collected with an isolation window of 13 m/z, a resolution of 30000, a maximum IT of 45 ms, a target value of 3e6 and 28% NCE. The 56 entries were collected in 12 Da windows starting at 367.5 m/z and ending with 1027.5 m/z.

Data analysis

To generate the spectral library for the DIA analysis the data from the HpH fractioned samples was first searched using MaxQuant version 1.5.3.17, against a mouse FASTA file downloaded in March 2018. The parameters used for the searches included carbamidomethylation of cysteines as a fixed modification and the variable modifications: methionine oxidation, protein N-terminal acetylation. Trypsin was used as the digestive enzyme, missed cleavages were set to a maximum of 2 and a reverse sequence database was used with a false discovery rate of 1%.

This generated list of proteins was used in the Skyline program for the DIA analysis to generate the spectral library. The same settings regarding modifications as in MaxQuant was used in Skyline, and the iRT peptides (Biognosys-11) was chosen as the retention time calibration/prediction standard, and the library was generated. Decoy peptides were generated using shuffled sequences. The DIA files were loaded into Skyline and searched against the spectral library. Peptides with no dotp score was removed, 1 % FDR was calculated and only peptide sequences with a Detection Q value below 0.01 was accepted in Skyline.

The generated list of proteins was further processed in Excel.

The intranasal administration of EVs

The intranasal administration of EVs were performed as described in the paper of Hu et al [3]. In short: mice were anesthetized with ketamine and xylazine. EVs were given alternatively in drops (2 µl per drop) to the nasal cavity. Absorbance of the previous drop

was observed before administrating the next drop of EVs. The dose of EVs was 1e+11 particles in 20 µl PBS per mouse. At 1h and 4h after EV injections, mice were sacrificed with an overdose pentobarbital and the brain was taken out.

1. Akimov, V.; Barrio-Hernandez, I.; Hansen, S.V.; Hallenborg, P.; Pedersen, A.-K.; Bekker-Jensen, D.B.; Puglia, M.; Christensen, S.D.; Vanselow, J.T.; Nielsen, M.M.J.N.s.; et al. UbiSite approach for comprehensive mapping of lysine and N-terminal ubiquitination sites. 2018. ***25****(7)*: p. 631-640.
2. Ægidius, H.M.; Veidal, S.S.; Feigh, M.; Hallenborg, P.; Puglia, M.; Pers, T.H.; Vrang, N.; Jelsing, J.; Kornum, B.R.; Blagoev, B.J.S.R. Multi-omics characterization of a diet-induced obese model of non-alcoholic steatohepatitis. 2020. ***10****(1)*: p. 1-12.
3. Hu, G.; Liao, K.; Niu, F.; Yang, L.; Dallon, B.W.; Callen, S.; Tian, C.; Shu, J.; Cui, J.; Sun, Z.; et al. Astrocyte EV-Induced lincRNA-Cox2 Regulates Microglial Phagocytosis: Implications for Morphine-Mediated Neurodegeneration. *Mol Ther Nucleic Acids* 2018. ***13***: p. 450-463.

Supplementary Tables

Table S1: List of qPCR primers used

Gene name	Forward primer (written 5′-3′)	Reverse primer (written 5′-3′)
IL-1β	CAGGCTCCGAGATGAACAAC	GGTGGAGAGCTTTCAGCTCATA
TNF-α	GTAGCCCACGTCGTAGGTAA	ATCGGCTGGCACCACTAGTT
GADPH	GACGGCCGCATCTTCTTGTG	GCGCCCAATACGGCCAAATC
HIF-1α	GATGACGGCGACATGGTTTAC	CTCACTGGGCCATTTCTGTGT

Table S2: Differentially expressed miRNAs in HR EVs compared to N EVs

miRNA	Normalized Counts	log2FoldChange	padj
mmu-miR-182-5p	33996	2.53	8.7003E-06
mmu-miR-183-5p	4155	2.71	0.0006
mmu-miR-25-3p	2010	1.92	0.0183
mmu-miR-486a-5p	25324	1.71	0.0208
mmu-miR-151-3p	6739	1.52	0.0317
mmu-miR-30a-3p	354	1.79	0.0567
mmu-miR-191-5p	6433	1.33	0.0729
mmu-miR-149-5p	1006	1.49	0.0729
mmu-miR-28a-3p	281	1.46	0.0729
mmu-miR-34c-5p	459	-2.50	0.0013
mmu-miR-423-3p	480	-1.43	0.0600
mmu-miR-744-5p	318	-1.66	0.0610
mmu-miR-125b-1-3p	842	-1.37	0.0729

Table S3: Differentially expressed miRNAs in HR C2C12 compared to N C2C12

miRNA	Normalized Counts	log2FoldChange	padj
mmu-miR-677-3p	289	4.116	4.8304E-06
mmu-miR-3084-3p	164	2.233	0.0050
mmu-miR-677-5p	384	2.062	0.0015
mmu-miR-3535	516	1.868	0.0804
mmu-miR-5099	1357	1.646	0.0576
mmu-miR-221-5p	1350	1.255	0.0582
mmu-miR-92a-1-5p	1250	-1.457	0.0046

Supplementary Figures

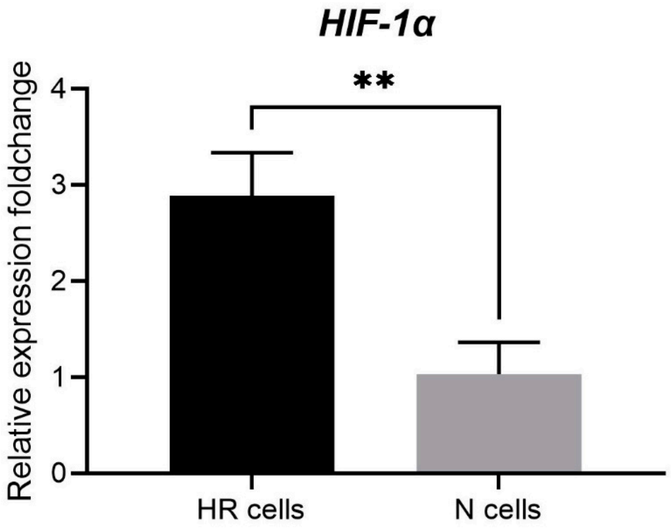

Supplementary Figure S1. QPCR quantification of *HIF-1 α* in C2C12 cells. *GADPH* was used to normalize the data and the fold-change is relative to N cells. N: normoxic cultured; HR: cyclic hypoxia-reoxygenation treated. The data is presented as mean ± SD; n =3 independent experiments; Unparied student t-test, ** p-value ≤ 0.01

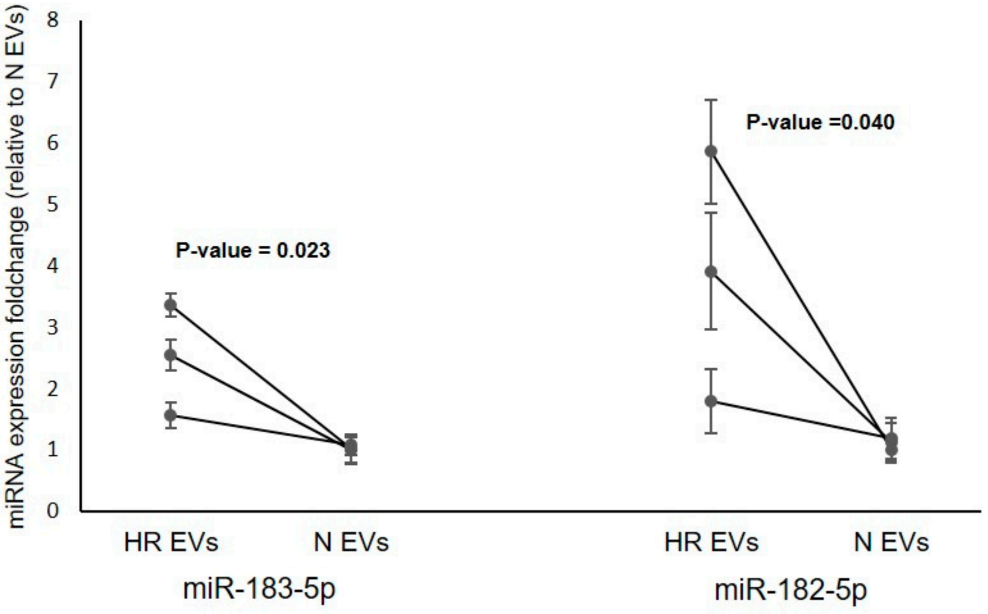

Supplementary Figure S2. Quantification of miR-182-5p and miR-183-5p in HR EVs and N EVs using Taqman miRNA assay. The same particle number of HR EVs and N EVs derived from the same batch were used for RNA purification and Taqman assay. The fold change of the miRNA expression was calculated by comparing HR EVs to N EVs from the same batch using $2^{-\Delta CT}$. The p-value was calculated using unpaired student t-test. The data is presented as mean ± SD; n =3 independent EV batches.

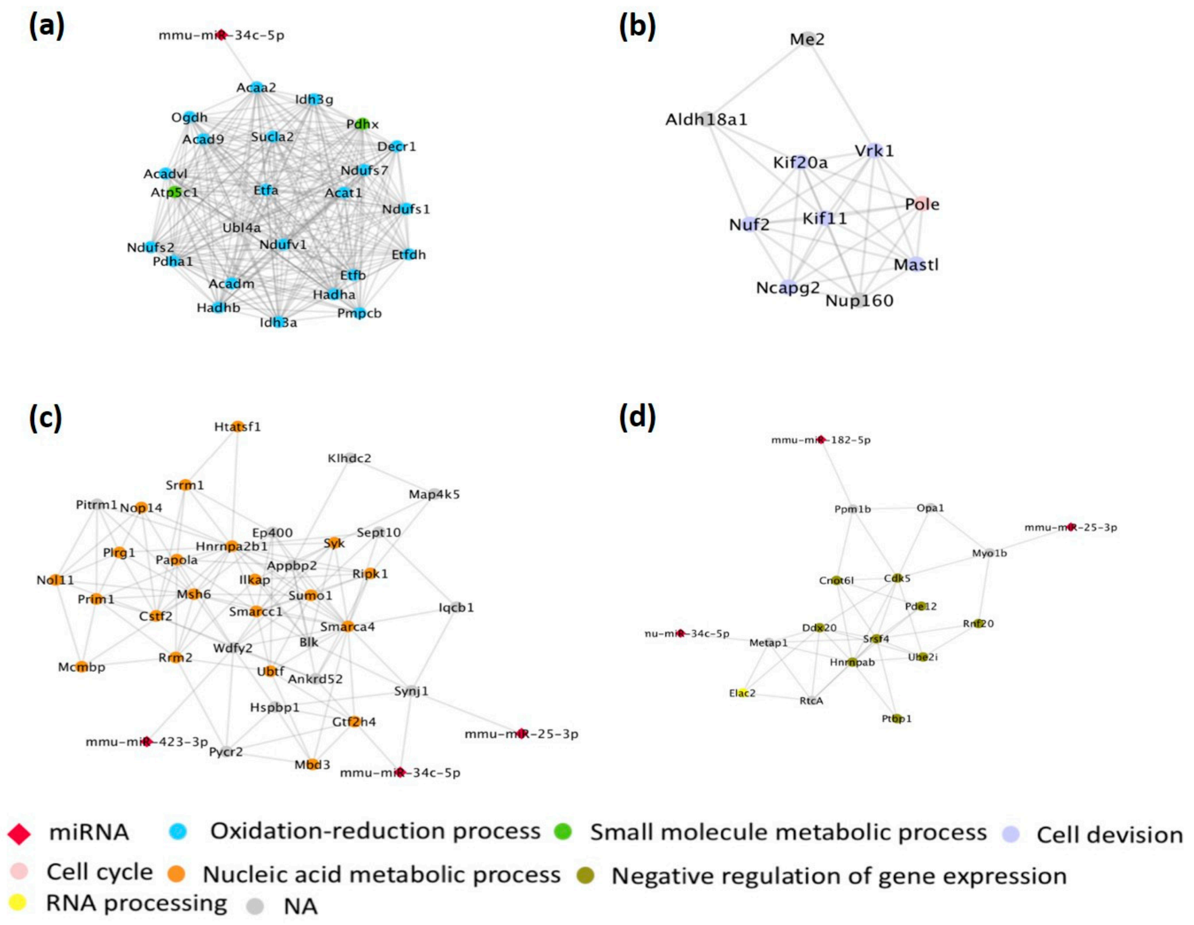

Supplementary Figure S3. The functional annotated subnetwork of computational protein-protein interaction analysis (PPI) of HR EV-specific proteins. The node colors are the enriched ontology term (biological pathway) at FDR ≤ 0.05 level. The grey nodes are no functional annotation or the annotated ontology term was below the significant threshold.

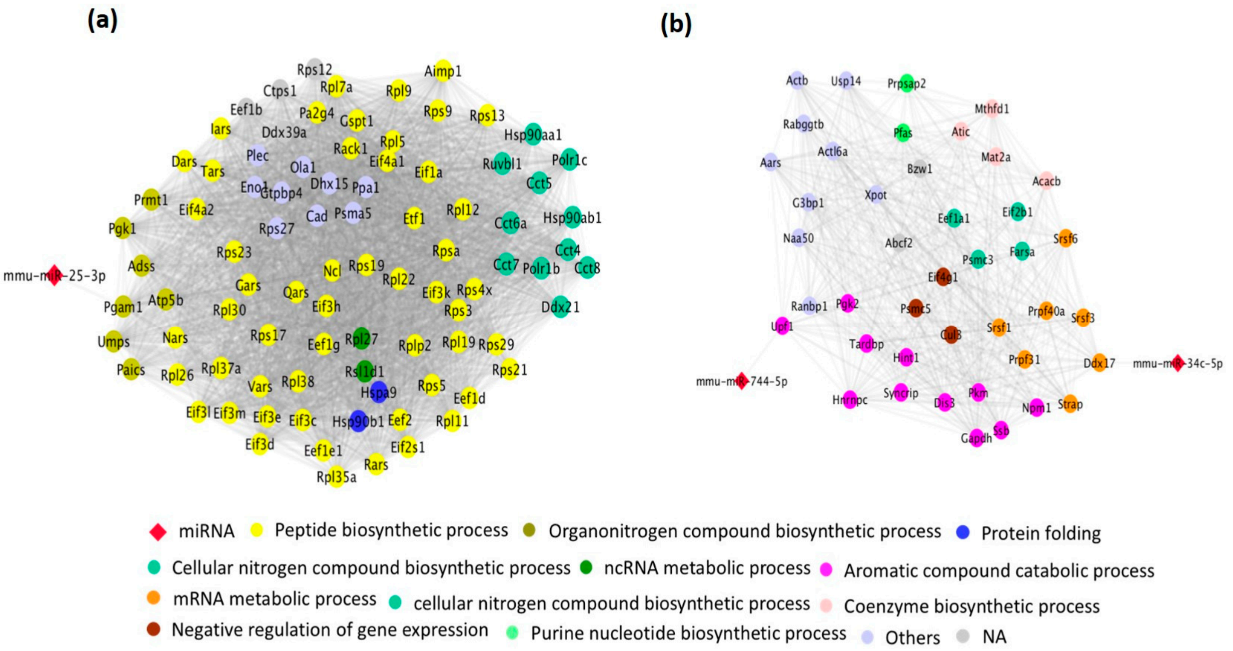

Supplementary Figure S4. The functional annotated subnetwork of computational protein-protein interaction analysis (PPI) of differentially expressed proteins in HR EVs. The node colors are the enriched ontology term (biological pathway) at FDR ≤ 0.05 level. The grey nodes are no functional annotation or the annotated ontology term was below the significant threshold.

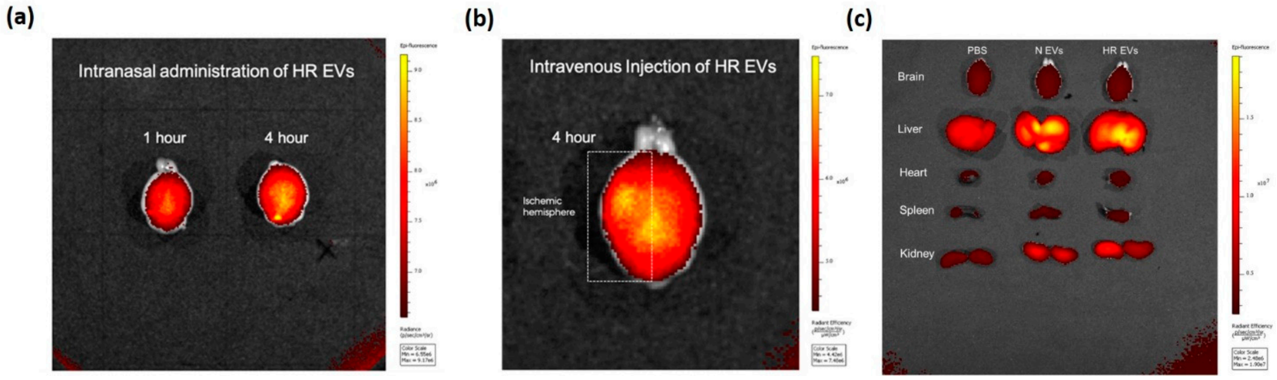

Supplementary Figure S5. The EV distribution in different organs using In Vivo Imaging System (IVIS) scanner. (a) The HR EV signal in brain after 1h and 4h intranasal administration. **(b)** The HR EV signal in brain after 4h intravenous injection. **(c)** The EV distribution in different organs 4h after intravenous injection. The EVs were labeled with a near-infrared dye. The mice were subjected to Transient Middle Cerebral Artery Occlusion (tMCAO) and 24h later the labeled EVs were injected.

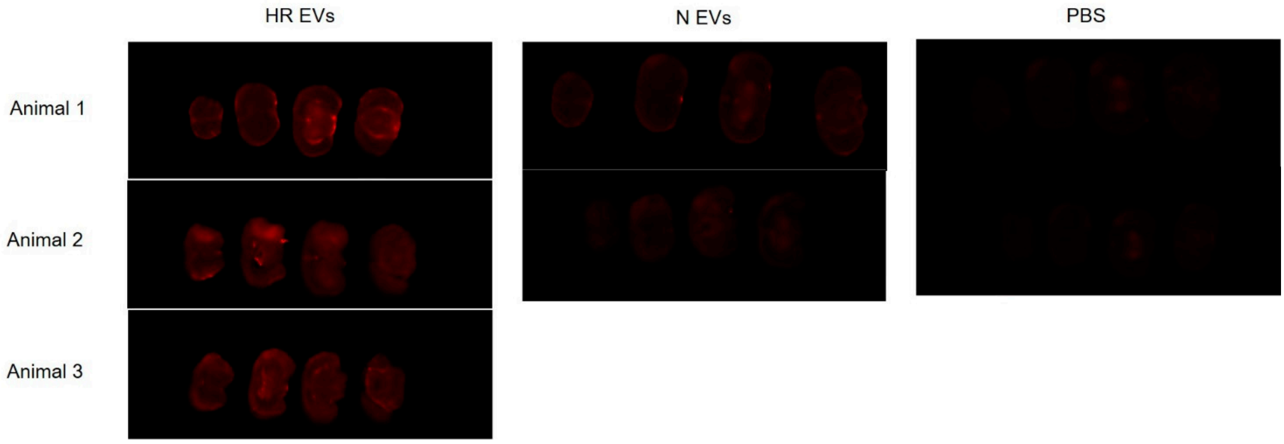

Supplementary Figure S6. Fluorescent images of brain sections from stroke mice injected with PBS, N EVs and HR EVs.

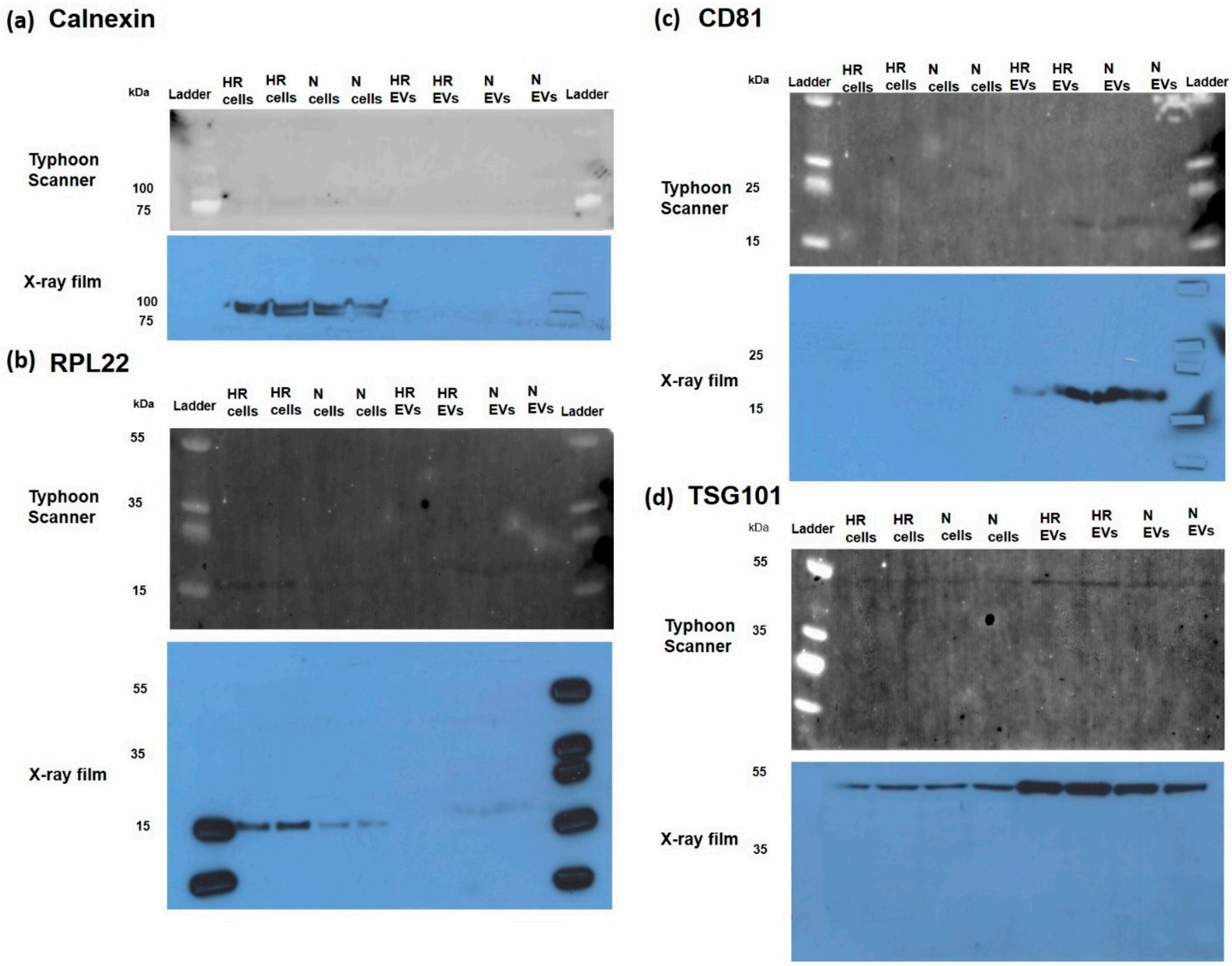

Supplementary Figure S7. Image of full-length Western blot for (a) Calnexin, **(b)** RPL22, **(c)** CD81 and **(d)** TSG101. Calnexin, RPL22 and CD81 detection was performed by probing different parts of same membrane using non-reducing protocol, while TSG101 detection was performed by probing on another membrane using reducing protocol. The grey image is obtained using typhoon scanner and the blue image is taken using X-ray films.
